# Mesenchymal stem cells-macrophages crosstalk and myeloid malignancy

**DOI:** 10.3389/fimmu.2024.1397005

**Published:** 2024-05-08

**Authors:** Kun Li, Hongyan Nie, Runming Jin, Xiaoyan Wu

**Affiliations:** ^1^ Department of Pediatrics, Union Hospital, Tongji Medical College, Huazhong University of Science and Technology, Wuhan, China; ^2^ Department of Radiology, Tongji Hospital, Tongji Medical College, Huazhong University of Science and Technology, Wuhan, China

**Keywords:** mesenchymal stem cells, macrophage, tumor microenvironment, immunomodulation, leukemia, lymphoma, myeloma

## Abstract

As major components of the tumor microenvironment, both mesenchymal stem cells (MSCs) and macrophages can be remodelled and exhibit different phenotypes and functions during tumor initiation and progression. In recent years, increasing evidence has shown that tumor-associated macrophages (TAMs) play a crucial role in the growth, metastasis, and chemotherapy resistance of hematological malignancies, and are associated with poor prognosis. Consequently, TAMs have emerged as promising therapeutic targets. Notably, MSCs exert a profound influence on modulating immune cell functions such as macrophages and granulocytes, thereby playing a crucial role in shaping the immunosuppressive microenvironment surrounding tumors. However, in hematological malignancies, the cellular and molecular mechanisms underlying the interaction between MSCs and macrophages have not been clearly elucidated. In this review, we provide an overview of the role of TAMs in various common hematological malignancies, and discuss the latest advances in understanding the interaction between MSCs and macrophages in disease progression. Additionally, potential therapeutic approaches targeting this relationship are outlined.

## Introduction

Mesenchymal stem cells (MSCs) are a type of non-hematopoietic stem cells that can self-renew and undergo multilineage differentiation. As an important component of the bone marrow microenvironment (BMME), MSCs play a crucial role in regulating the proliferation and differentiation of hematopoietic cells (1, 2).

Moreover, extensive research has indicated that MSCs significantly influence the growth of various tumors (3, 4) (including hematological malignancies) and actively participate in pathological and physiological processes, as well as disease progression in leukemia, multiple myeloma (MM), and other related disorders (5, 6). In addition to providing direct support to tumors, MSCs also possess a diverse range of immunomodulatory properties that can alter the local tissue microenvironment through direct cellular interaction or secretion of various immune-related molecules including transforming growth factor-β (TGF-β), stem cell growth factor (SCF), prostaglandin E2 (PGE2), and interferon-gamma (IFN-γ), thereby facilitating tumor immune evasion and progression (7, 8).

As a crucial component of the immune system, macrophages play a pivotal role in the tissue microenvironment and actively participate in tumor growth and metastasis (9–12). Due to their plasticity and diversity, macrophages have gradually gained recognition for their potential applications in the treatment of numerous diseases. However, macrophages within the tumor microenvironment (TME), which are also referred to as tumor-associated macrophages (TAMs), exhibit distinct phenotypic characteristics and immune functions that are primarily characterized by their immunosuppressive properties and promotion of tumor growth (10, 13). They impede the clearance of abnormal cancer cells by secreting immunosuppressive factors, weakening T lymphocyte activity, and interfering with other immune effector organs (14–17).

In recent years, there has been a growing focus on the study of MSCs and macrophages in inflammatory microenvironments, bone healing and gastrointestinal neoplasms (18–22). However, there is a dearth of research focusing on the impact of the interaction between MSCs and macrophages in the context of hematologic malignancies. This article reviews the intricate interplay between MSCs and macrophages within the microenvironment of myeloid malignancies.

## Biology of macrophages

Typically, macrophages originate from hematopoietic stem cells in the bone marrow and develop into monocyte progenitors under the stimulation of multiple colony-stimulating factor (multi-CSF) and granulocyte-macrophage colony-stimulating factor (GM-CSF) (23, 24). Monocyte progenitors further differentiate into premonocytes and enter the bloodstream, where they differentiate into mature monocytes and eventually migrate to tissues to differentiate into tissue-specific macrophages. These include the skeletal system (osteoclasts), the central nervous system (microglia), the lungs (alveolar macrophages), the liver (Kupffer cells), and connective tissue (histiocytes), as well as the spleen, gastrointestinal tract, and peritoneum (25, 26). In recent years, with the development of lineage tracing technology, it has been found that microglia and some other tissue-resident macrophages originate from the yolk sac during embryonic development independently of hematopoietic stem cells (24).

Macrophages play an indispensable role in immune and inflammatory processes due to their plasticity and diversity (13, 27, 28). In addition to their well-known phagocytic function and antigen-presenting ability, macrophages possess other important functions. First, as key participants in regulating immune responses, they can secrete various cytokines such as tumor necrosis factor-α (TNF-α), interleukin-1 (IL-1), IL-6, IL-12, IL-10, transforming growth factor-β (TGF-β) and chemokines (CXCL8 and CCL5), to modulate the activities of other immune-related cells in the immune system (29, 30). These secreted molecules can activate other immune cells and promote effective local tissue defence reactions. Second, macrophages are also involved in tissue repair and regeneration processes (29, 30). In summary, macrophages within wounds exhibit anti-inflammatory properties by producing anti-inflammatory factors such as IL-10 and arginase 1 (Arg1), as well as TGF-β, vascular endothelial growth factor (VEGF), and EGF. This promotes the resolution of inflammation in the wound and facilitates tissue remodelling, fibrosis, and healing.

In response to various environmental stimuli, particularly under pathological conditions, macrophages exhibit diverse functions and morphologies known as macrophage polarization (31, 32). This crucial immune response process plays a pivotal role in the defence against infections and elimination of aberrant cells. Activated macrophages are divided into two major phenotypes:classically activated macrophages (M1) and alternatively activated macrophages (M2) (32). In simple terms, M1 macrophages are stimulated with lipopolysaccharide (LPS), interferon-gamma (IFN-γ), GM-CSF, or TNF-α. They are characterized by the secretion of cytokines such as IL-1β, IL-6, IL-12 and TNF-α. In addition, they express high levels of major histocompatibility complex class II (MHC-II), CD68 markers and the costimulatory molecules CD80 and CD86. Studies have shown that M1 macrophages can activate inducible nitric oxide synthase (iNOS) to produce NO by upregulating the expression of intracellular suppressor of cytokine signalling 3 (SOCS3). Thus, M1 macrophages exhibit proinflammatory or antitumor effects (33). In contrast, M2 macrophages activated by IL-4, IL-13, or CSF-1 can express Arg, TGF-β, IL-10, metalloproteinases, and other factors, thus playing important roles in tissue repair, immune suppression, or angiogenesis (33–35). In fact, M2 can be further divided into four types: M2a, M2b, M2c and M2d. M2a macrophages are induced by IL-4 and IL-13 and express high levels of mannose receptor (CD206), IL-1 receptor antagonist (IL1Ra), and Arg1. IL1Ra can inhibit the generation of M1 macrophages and has an anti-inflammatory effect. Arg1 activates the arginine pathway to produce ornithine, which is a precursor of polyamines and collagen that contributes to the production of extracellular matrix and promotes tissue repair. M2b is induced by immune complexes and some Toll-like receptor (TLR) agonists, such as LPS, and simultaneously produces anti-inflammatory and pro-inflammatory cytokines, such as IL-10, IL-1β and IL-6. M2c is induced by glucocorticoids and IL-10, which release a large amount of IL-10, downregulate the production of inflammatory cytokines and inhibit the immune response. It produces profibrotic TGF-β, which plays an important role in the process of tissue remodelling. Finally, M2d macrophages, which are induced synergistically by Toll-like receptor (TLR) agonists and adenosine receptor agonists, secrete IL-10 and VEGF, thereby promoting angiogenesis and exhibiting a strong association with tumor growth. The latest review by Lazarov et al. provides comprehensive insights into the origin, characteristics, and functions of macrophages (36).

In addition to M1 and M2 macrophages, there is another subgroup called TAMs. TAMs are generally considered M2-like macrophages, which play a role in promoting tumor cell proliferation, survival, infiltration and metastasis, as well as facilitating tumor angiogenesis (13, 37). However, there is still ongoing debate regarding the phenotypic characteristics of TAMs and their functions within the TME. Some studies have demonstrated that TAMs exhibit gene expression profiles resembling those of both M1 and M2-like macrophages (38, 39). The presence of M1 and M2 TAMs has been observed throughout all stages of tumor development. During the early stage, TAMs predominantly exhibit a proinflammatory and antitumor phenotype (M1). However, as the tumor progresses, TAMs gradually transition towards an anti-inflammatory and protumor phenotype (M2), with an increased abundance of M2 TAMs indicating a poor prognosis. However, although this M1-M2 paradigm is dominant in TAMs biology, it is an oversimplification that fails to describe the multitude of macrophage states in tumors.

**Figure 1:**
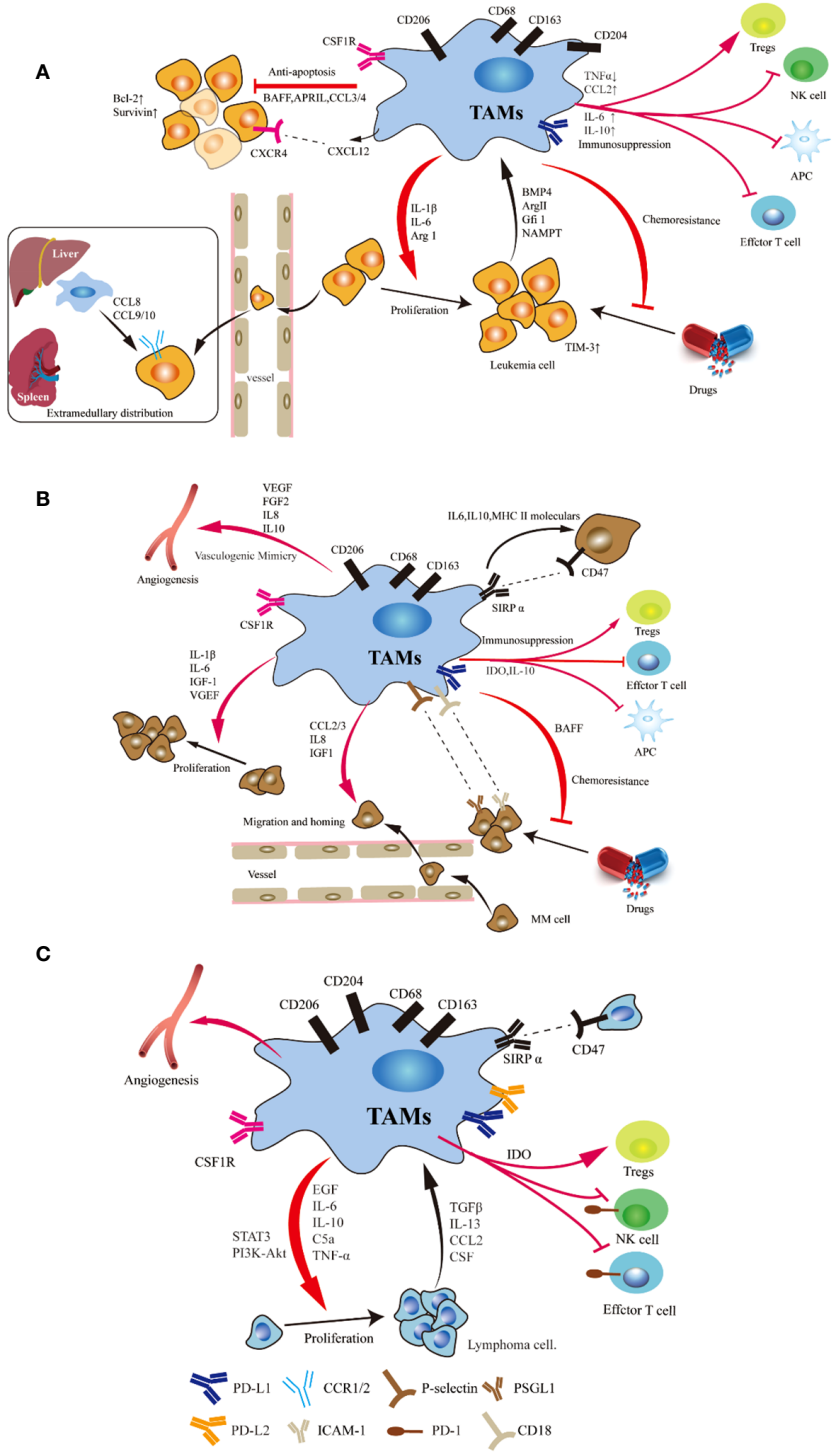
Schematic representation of the signals involved in the crosstalk between tumor cells and TAMs in leukemia **(A)**, myeloma **(B)** and lymphoma **(C)**. Studies have demonstrated the significant impact of macrophages on various crucial pathways involved in the initiation and progression of leukemia, myeloma and lymphoma, including anti-apoptosis and proliferation, immunosuppression, angiogenesis, extramedullary migration, tumor cell homing and drug resistance. Tumor cell can induce a shift in monocyte polarization towards an inhibitory M2-like phenotype (mainly characterized by upregulated CD206 and CD163 expression), thereby promoting their own survival.

Currently, an increasing number of scholars contend that TAMs do not exist in a stable form but rather exhibit distinct M1 or M2 phenotypes under the influence of external factors (13, 40). Furthermore, both subsets of macrophages coexist within tumor tissues and exert dual effects on tumor growth. In recent years, research on macrophages has focused predominantly on solid tumors, whereas investigations into hematological malignancies have been relatively scarce. The subsequent discussion will delve into a more comprehensive exploration of the role of macrophages in hematologic malignancies (**Figure 1**).

## Macrophages in leukemia

In contrast to solid tumors, hematological malignancies exhibit a unique immune microenvironment. TAMs within the leukemia microenvironment are also referred to as leukemic-associated macrophages (LAMs). In recent years, there has been an increasing focus on the role of LAMs in various types of leukemia, including acute lymphoblastic leukemia (ALL), acute myeloid leukemia (AML), chronic lymphocytic leukemia (CLL), and chronic myeloid leukemia (CML) (9, 41–44).

### Acute lymphoblastic leukemia

The role of TAMs in adult T-cell leukemia/lymphoma (ATLL) was first proposed by Komohara (45), which led to beginning of research on macrophages in leukemia. This study demonstrated that the proportion of CD163^+^ macrophages is associated with clinical prognosis and that this proportion is an independent factor influencing prognosis. Through *in vitro* co-culture, it was observed that CD163^+^ M2 macrophages significantly promoted the proliferation of leukemia cells. The study also demonstrateda significant positive correlation between CD204^+^ TAMs and the Ki-67 labeling index in ATLL, thus suggesting the potential involvement of CD204^+^ TAMs in ATLL cell proliferation (46). To elucidate the intricate mechanisms underlying chemoresistance in ATLL cells, an inhibitor targeting M-CSFR was used to inhibit the interaction between macrophages and leukemia cells (47, 48). The findings demonstrated that administration of the M-CSFR inhibitor triggered apoptosis in ATLL cells while concurrently suppressing PD-1 ligand expression in lymphoma cells and macrophages.

There is evidence of organ and temporal specificity of macrophages in a Notch1-induced mouse model of T-ALL. Their distribution and phenotype can differ in the bone marrow, spleen and liver during the development of leukemia (49–51). LAMs have been shown to stimulate T-ALL cell proliferation and enhance the migratory activity of ALL cells. Furthermore, LAMs in the spleen can exert a more pronounced proliferative effect than those in the bone marrow. In addition, researchers also analyzed the subsets of hepatic and peritoneal macrophages and the expression of phenotype-related genes in T-ALL mice. In hepatic LAMs, the majority of M1-associated genes, including CCL5, CXCL1, IL-12β, iNOS, and TNF-α, exhibit increased expression during leukemia progression. Conversely, the expression of CXCL11 and IL-1β decreased. Furthermore, the expression of most M2-associated genes, such as Arg1, CCL3, CCL17, CD206, IL-10, and TGF-β decreases with the infiltration of leukemia cells, whereas the expression of MMP9 and VEGFα increases in the advanced stages of leukemia. Additionally, in peritoneal LAMs, the expression of CD206, CXCL9 and iNOS initially increases and subsequently decreases as the tumor progresses, whereas the expression of IL-1β and IL-6 first decreases and then increases. These findings suggest that the functional and phenotypic characteristics of LAMs are influenced by tissue-specific microenvironments.

The B-cell leukemia cell line Nalm-6 can enhance the generation of immunosuppressive dendritic cells and shift M1-like macrophages towards a less proinflammatory phenotype through bone morphogenetic protein 4 (BMP4) signaling (52). Similarly, overexpressing BMP4 in ALL cells enhances the ability of these cells to induce immunosuppressive dendritic cells and favors the generation of M2-like macrophages with pro-tumor characteristics.

To further understand the effect of the leukemia microenvironment on the gene expression of LAMs, hierarchical clustering analysis was used to analyze the gene expression characteristics of LAMs in the bone marrow and spleen of T-ALL and AML (53). The findings demonstrated that metabolic pathways and the cell cycle were enriched in LAMs from the bone marrow and spleen of mice with T-ALL. By analyzing various genes associated with the M1/M2 phenotype, including iNOS, TNF-α, CXCL10, CXCL9, TGF-β1, IL-12β and IL-6, researchers found that spleen LAMs exhibited more M2 characteristics in T-ALL and AML mice, whereas bone marrow LAMs displayed more M1 features. Further investigation revealed that the IRF7-SAPK/JNK pathway, rather than the STAT1 or STAT6 pathway, was crucial for the different polarization of LAMs. Therefore, the targeting of the IRF7-SAPK/JNK pathway to induce M1-like characteristics may contribute to prolonging survival in leukemia mice. However, further validation is required to determine whether the activation of the IRF7-SAPK/JNK pathway also leads to increased M1 characteristics in human macrophages.

Hohtari et al. provided a comprehensive characterization of the immune cell composition within the BMME at the time of adult B-cell precursor ALL diagnosis (54). The proportion of M1 macrophages in the bone marrow of ALL patients was lower than that in healthy controls; in contrast, the proportion of M2 macrophages was greater.

By utilizing a Notch1-induced model of T-ALL in wild-type (WT) and CCR2^-/-^ mice, Yang et al. elucidated that LAMs originate from peripheral blood mononuclear cells (PBMCs) (55). Furthermore, they highlighted the role of the leukemic microenvironment in recruiting an increased number of monocyte-derived LAMs with more M1-associated features. In the early stages of leukemia, a greater abundance of LAMs was observed within the liver and spleen microenvironment. Subsequent investigations demonstrated that CCR2^-/-^ leukemia mice exhibited a significant reduction in peripheral blood leukemia cells, although no discernible difference was detected in the bone marrow. Additionally, CCR2^-/-^ leukemia mice displayed less hepatosplenomegaly and fewer leukemia cells compared to WT mice. Further comprehensive studies revealed that LAMs facilitate the extramedullary distribution of leukemia cells through modulation of the CCL8/CCL9-CCR1/CCR2 axis (55). Importantly, blockade of both the CCR1 and CCR2 receptors effectively inhibited the extramedullary infiltration of leukemia cells while alleviating hepatosplenomegaly symptoms.

The aforementioned studies provide valuable insights into the role of LAMs in ALL.However, further comprehensive investigations are warranted to elucidate the underlying regulatory mechanisms between leukemia cells and macrophages.

### Acute myeloid leukemia

Approximately ten years ago, Mussai et al. initially elucidated multiple mechanisms through which AML exerts immunosuppressive effects (56). Initially, they reported the ability of AML blasts to inhibit the growth of murine and human hematopoietic precursors *in vitro*, thus revealing that this effect was mediated by the secretion of the arginase II enzyme. Further investigation demonstrateda significant enrichment of CD206^+^ monocytes in the BM of newly diagnosed AML patients (56, 57). Moreover, studies have demonstrated that high infiltration of M2 macrophages is significantly associated with an unfavorable prognosis in AML patients (56–60). Thus, CD206, which is a marker gene of M2 macrophages, has the potential to emerge as a novel prognostic indicator for AML. In two independent cohorts of AML patients, elevated expression levels of CD206 were indicative of poor overall survival (OS) and event-free survival (EFS) (56, 58). Similarly, they observed a significant upregulation of YM-1, which is a mouse M2 marker, in Ly6C^+^ monocytes derived from the BM of NOD-SCID mice that were transplanted with human AML blasts (56). Co-culture experiments demonstrated that AML cells and their culture supernatant could induce a shift in monocyte polarization towards an inhibitory M2-like phenotype (characterized by upregulated CD206 and CD163 expression), consequently suppressing T-cell proliferation and establishing an immunosuppressive microenvironment (56, 59, 61). These effects were found to be dependent on arginase II activity, as the administration of inducible nitric oxide synthase (iNOS) inhibitors (NOHA and L-NMMA) or exogenous arginine significantly attenuated CD206 expression in co-cultured macrophages while restoring T-cell proliferation (56). Collectively, these findings confirm the capacity of AML blasts to generate an immunosuppressive microenvironment within the BM and PB, thereby promoting their own survival (53, 56, 61).

Subsequent investigations revealed that AML induces the infiltration of AML-associated macrophages (AAMs) into the bone marrow and spleen of mice, thereby promoting their proliferation and accumulation in recipient mice (57). Similarly, there was a significant increase in the proportion of CD163^+^CD206^+^ AAMs in the bone marrow of AML patients. Compared with those derived from nonleukaemic mice, bone marrow-derived macrophages (BMDMs) derived from different leukemia mice exhibited an enhanced ability to expand AML cells *in vitro* (57). The extent of macrophage infiltration *in vivo* was correlated with the survival rate of the mice. They found that growth factor independence 1 (Gfi1), which is a transcriptional repressor, plays a crucial role in macrophage polarization and that its loss prevents macrophage polarization to a pro-leukemic state, thus resulting in an antitumor state, both *in vitro* and *in vivo* (57). The findings presented in this study offer novel insights into potential therapeutic strategies for the treatment of AML.

To elucidate the potential role of nonmalignant macrophages derived from bone marrow in promoting chemotherapy resistance in AML blasts, researchers have discovered that the majority of granulocytes (CD11b^+^F4/80^-^Ly6G^+^) and monocytes (CD11b^+^F4/80^+^CD169^-^) were eliminated following treatment with cytarabine and doxorubicin (60). However, a subset of macrophages characterized by CD11b^-^F4/80^+^CD169^+^VCAM1^+^ expression remained resilient to chemotherapy within the BMME. To further investigate the possible role of these macrophages in the response of AML blasts to chemotherapy, specific depletion of CD169^+^ macrophages was conducted in mice followed by treatment with cytarabine and doxorubicin (60). The results showed that leukemia mice depleted of CD169^+^ macrophages exhibited significantly prolonged median survival compared to wild-type mice, which was accompanied by notable reductions in leukemia burden within both the blood and spleen. Furthermore, AML patients with high expression of CD163 in macrophages have a shorter survival period than those with low expression of CD163, whereas the expression of CD68 has no significant effect on survival time (53). This finding suggested that M2 macrophages, rather than total macrophages, may contribute to a poor prognosis in AML patients. Macrophages in the BM and spleen exhibit heterogeneity under different microenvironmental influences. Specifically, compared with BM macrophages, spleen macrophages exhibit more functional characteristics associated with the M2 phenotype (53). Furthermore, the level of IRF7 expression was greater in BM LAMs than in spleen LAMs, thus indicating that IRF7 may be responsible for the phenotypic differences between BM and spleen LAMs. Similarly, in MLL-AF9-induced AML mice, peritoneal resident macrophages also displayed an M2 phenotype (62). Subsequent studies have confirmed that IRF7 can activate the SAPK/JNK pathway in macrophages to promote their polarization towards the M1 phenotype, thereby prolonging the survival time of leukemia mice. Several studies have also demonstrated that the utilization of a CSF1R inhibitor in combination with GM-CSF effectively induces the polarization of pro-tumor M2 macrophages towards anti-tumor M1 macrophages in the TME, thus reversing TAMs-induced tumor resistance and promoting apoptosis of myeloblasts (61, 63). Moore et al. initially proposed that LC3–associated phagocytosis of BM macrophages serves as a major mechanism for mediating the apoptosis of AML cells and can activate the stimulator of IFN genes (STING) pathway in macrophages, thus enhancing their phagocytic capacity and effectively suppressing AML growth (64). These findings suggest that the elimination of macrophages may lead to an increase in tumor burden in AML, which seems to contradict the findings of previous studies. The observed inconsistency may be attributed to the presence of diverse macrophage subtypes in AML, each exhibiting distinct mechanisms of action. Macrophages derived from AML exhibit immunosuppressive and leukemogenic functions (59). Researchers have observed significant heterogeneity in macrophages among AML patients, with a poor prognosis associated with a high abundance of M2 macrophages. Furthermore, macrophages are capable of driving the invasion of primary AML and acute promyelocytic leukemia (APL) cells. These findings demonstrate that AML-derived M2 macrophages drive leukemic transformation by evading phagocytosis and enhancing mitochondrial metabolism. Therefore, the targeting of mitochondrial energy production in M2 macrophages could impair their capacity to support AML blasts. These results further highlight the heterogeneity of the macrophage sub-population in AML.

Monocytic leukemia zinc-finger protein (MOZ) has been shown to play an important role in the development and maintenance of hematopoietic stem cells (HSCs), and its expression is significantly reduced in patients with acute monocytic leukemia (AMoL) (65). Further experiments showed that MOZ promoted the differentiation of monocytes into macrophages and M1 polarization (65). In addition, MOZ knockdown increases the resistance of AMoL cells to chemotherapy-induced apoptosis and is closely associated with an unfavorable prognosis for AMoL patients.

### Chronic lymphocytic leukemia

TAMs in CLL are commonly referred to as nurse-like cells (NLCs) because they have functions and characteristics similar to those of nurse cells and exhibit high expression levels of CD68 and CD163 (66–70). The number of CD68-positive NLCs was significantly greater in the lymph nodes of CLL patients than in those of controls, and increased CD68 expression was associated with shorter overall survival (71)..

NLCs have been found to play a significant role in the development and progression of CLL. These specialized cells, which are typically present in the BMME, provide crucial support to CLL cells by promoting their survival and protecting them from chemotherapy-induced cell death (72).

Due to the expression of CD68 in non-myeloid cells, Boissard et al. believe that using CD68 may overestimate the exact number of NLCs (72). Therefore, they decided to use CD163 as the most relevant marker for NLCs. They observed a significant increase in the abundance of CD163^+^ cells within the TME during chemotherapy, and there was a strong correlation between elevated serum levels of soluble CD163 and unfavorable prognostic markers. The findings from this study demonstrate, for the first time, that aggressive CLL modulates the TME to enhance monocyte differentiation into CD163^+^ cells.

Research has shown that NLCs from the blood of CLL patients can activate the p44/42 MAPK signaling pathway within CLL cells through the SDF-1α/CXCR4 pathway, thereby protecting CLL B cells from apoptosis and promoting their survival *in vitro* (73). When co-cultured with NLCs, CLL lymphocytes were not only able to effectively escape spontaneous apoptosis; however, the presence of NLCs significantly reduced the apoptosis induced by ibrutinib, dexamethasone and chlorambucil (67, 74). Intriguingly, under NLC-depleted culture conditions, most CLL lymphocytes died within 10 days. However, when co-cultured with NLCs, CLL cells survived for up to 14 weeks (74). The investigators observed an increasein the expression of genes encoding anti-apoptotic proteins such as BCL2 and SURVIVIN in co-cultured CLL lymphocytes. Additionally, these cells exhibited alterations in gene expression related to cell cycle regulation, differentiation and transcription processes. Conversely, genes associated with promoting apoptosis, as well as growth factor/chemokine receptors were downregulated in the context of co-culture (74). The interaction between NLCs and CLL has been extensively evaluated, thus demonstrating that CLL cells stimulate the differentiation of NLCs, whereasNLCs reciprocally secrete factors that recruit CLL cells and enhance their survival (71, 75, 76). NLCs protect CLL cells against apoptosis through various cytokines, including a proliferation inducing ligand (APRIL), B-cell activating factor (BAFF), CXCL12, CD31, Plexin B1, CCL3 and CCL4 (73, 77–80). This crosstalk appears to be independent of direct cell-cell contact, which is not required for pro-survival signaling in NLCs (66, 74, 77). However, it should be noted that it has been proposed that *in vitro* CD14^+^ cell differentiation into NLCs requires contact with CLL cells (68), which seems to contradict the findings of previous studies. Future studies are needed to gain insight into the mechanisms underlying the behavior of NLCs. Targeting therapies against these cells will hopefully overcome the pro-survival effects of the TME in CLL patients (81, 82).

### Chronic myeloid leukemia

K562-derived exosomes can significantly up-regulate the mRNA levels of TNF-α and IL-10 in macrophages, significantly downregulate the mRNA level of the INOS gene, and promote the polarization of macrophages to TAMs (83).

The expression levels of CD68, CD163 and CD206 in the bone marrow of CML patients were significantly elevated compared to those in the control group (84). Moreover, the positive expression of these molecular gradually increased concomitant with disease progression. CML red pulp macrophages (RPMs) are able to produce a variety of cytokines and chemokines such as IL-10, CCL3, CCL4, CCL5, CXCL1, TNF-α, and SCF, to sustain leukemia initial cells (LICs) (85). RPMs constitute the microenvironment of splenic leukemia and induce LICs quiescence. The depletion of macrophages leads to reduced spleen weight, diminished total leukemic cell count, and significantly decreased spleen leukemia burden. *In vitro* experiments have demonstrated that RPMs substantially enhanced the colony formation ability of LICs while reducing their sensitivity to imatinib treatment.

Macrophages can protect CML cells from natural killer (NK) cell attack in a state of chronic infection and inflammation. The co-culture of macrophages (Mφ), NK cells, and CML cells was employed to elucidate the regulatory effects exerted by macrophages and NK cells on CML cells activity. The data revealed an upregulation in the expression of CD107a, which is a marker indicative of NK cells activation, within the NK+CML co-culture system, thereby contributing to a reduction in the CML survival rate. Moreover, the proportion of CD107a^+^ NK cells within the Mφ+NK+CML co-culture system was lower than that within the NK+CML co-culture system, thus suggesting that macrophages inhibit NK cells degranulation and cytotoxicity and consequently promote CML cells survival. Ultimately, research has confirmed that macrophages exert a protective effect on CML by modulating the expression levels of CD16 on NK cells membranes, thereby inhibiting NK cells degranulation and cytotoxicity (86).

## Macrophages in multiple myeloma

At present, the role of TAMs in the growth of MM has been extensively explored (87–89). Numerous studies have demonstrated the significant impact of macrophages on various crucial pathways involved in the initiation and progression of MM, including tumor cell homing (90, 91), proliferation and survival (92, 93), immunosuppression (94, 95), angiogenesis (96, 97), and drug resistance (98, 99). MM cells can secrete various chemokines, such as CCL2, CCL5, CXCL1, CXCL12 and CXCL13, which specifically recruit peripheral blood monocytes to promote their homing, proliferation, and polarization (100, 101). CD206^+^MERTK^+^ M2-like macrophages were significantly increased in the bone marrow of MM mice, leading to a significant increase in CXCL13 levels (100). Specifically, MM cells upregulate the expression of CXCL13 in macrophages through the BTK signaling pathway, while macrophages promote the expression of CXCL13 within MM cells through the TGF-β signaling pathway (100). High levels of CXCL13 can promote the proliferation of MM cells and the polarization of M2 macrophages and are potentially associated with extramedullary involvement in MM. Li et al. performed single-cell RNA sequencing on samples from various stages of MM and characterized the reprogramming of macrophages during disease progression (102). They also emphasized the strong correlation between CD47 and macrophage inhibitory factor (MIF) expression with disease advancement and unfavorable prognosis. By employing a dual-targeting TAMs strategy involving anti-CD47 antibodies and MIF inhibitors, they successfully stimulated phagocytosis and facilitated TAMs repolarization, thereby exerting potent anti-tumor effects.

Research findings have indicated that the accumulation of total CD68^+^ macrophages and the upregulation of CD206^+^ M2 macrophages in the bone marrow are associated with an unfavorable prognosis in patients with MM (103, 104). Furthermore, the clustering of CD163^+^ M2 macrophages is also correlated with shorter overall survival and is considered an independent adverse prognostic factor (105). Subsequent studies have corroborated these results, as elevated levels of soluble CD206 (sCD206) and CD163 (sCD163) in serum also serve as indicators of poorer overall survival (106, 107).

MM is an incurable cancer that relies on signals obtained from the BMME to promote survival and proliferation (108, 109). Initially, IL-6 was believed to be secreted by malignant plasma cells (110). However, subsequent research has indicated that IL-6 primarily originates from BM-MSCs within the niche and plays a crucial role in the survival and proliferation of MM cells (93, 111). Kim et al. identified macrophages as being a supplementary source of IL-6, thus providing an optimal environment for tumor cell growth (93). IL-6 is a pleiotropic inflammatory factor that is associated with poor prognosis of MM (112, 113). Studies have shown that IL-6 can promote MM cell proliferation and inhibit apoptosis through the Ras/Raf/MAPK and PI3K/AKT pathways in MM cells, and induce osteoclast generation through the JAK2/STAT3 axis.

In addition to promoting proliferation, TAMs also facilitate chemoresistance in MM. After co-culturing with different MM cell lines, it was found that both MSCs and macrophages effectively inhibited the apoptosis of MM cells (93). However, when co-cultured with MSCs alone, there was no significant change in the apoptosis level of primary MM cells. In contrast, when co-cultured with macrophages or simultaneously with MSCs and macrophages, the degree of apoptosis in primary MM cells significantly decreased. The interaction between macrophage selectin and its ligand PSGL-1 promotes melphalan resistance in MM cells (98). Additionally, ICAM-1/CD18 are crucial molecules involved in mediating chemoresistance (98). The phosphorylation of Src and Erk1/2 kinases and the activation of the C-myc pathway enable macrophages to promote the drug resistance of MM cells.

Bortezomib, which is a pioneering proteasome inhibitor, has emerged as a widely employed therapeutic agent for the treatment of MM and mantle cell lymphoma (MCL) in clinical settings (104, 114). However, recent studies have shown that some patients develop resistance to bortezomib, which is due to the release of IL-1β by TAMs, leading to an increase in the number of MM initiating cells (104). In addition, macrophages promote the survival of MM cells by activating the NF-κB pathway through the expression of BAFF, thereby preventing bortezomib-induced apoptosis.

In fact, an increasing body of research has demonstrated that MM-associated macrophages predominantly exhibit M2-like characteristics and exert immunosuppressive effects by inhibiting T-cell proliferation, thereby facilitating immune evasion of MM cells (115–117). As antigen presenting cells (APCs), M1 macrophages express high levels of major histocompatibility complex class II (MHC II), thus facilitating more efficient pathogen recognition by the adaptive immune system. However, due to the dysregulation of MHC II expression in MM-associated macrophages, T-cell function is suppressed, thereby weakening the anti-tumor response (94). IL-10, a crucial immunosuppressive cytokine primarily secreted by TAMs, can inhibit MHC II molecule expression and pro-inflammatory cytokine production, thus effectively suppressing cytotoxic T-cell activation (117–119). In addition to its immunosuppressive effects, IL-10 also plays a pivotal role in the survival, proliferation, and angiogenesis of patients with MM (96). Elevated levels of IL-10 in serum have been associated with an unfavorable prognosis in MM patients. In addition, macrophages in MM suppress the proliferation and activation of T cells by inhibiting the secretion of IFN-γ (115). In summary, macrophages associated with MM secrete a variety of signaling molecules that exert inhibitory effects on T-cell function (93, 94, 120).

Accumulated evidence suggests that angiogenesis plays a crucial role in the development and adverse prognosis of MM. Macrophages are a source of pro-angiogenic factors, such as vascular endothelial growth factor (VEGF), fibroblast growth factor 2 (FGF 2) and IL-8, which can promote neovascularization and vascular remodeling (121, 122). When treated with VEGF and FGF, macrophages derived from the TME exhibit a vascular, endothelial phenotype. In addition, IL-10 derived from TAMs is positively correlated with angiogenic factors such as VEGF, angiopoietin-2, and proliferation markers. Zhang and colleagues discovered that B-cell specific moloney murine leukemia virus integration region 1 (BMI1) is upregulated in macrophages of MM (123). BMI1, a member of polycomb-group proteins, is directly involved in the regulation of cell growth and proliferation and is required for the self-renewal of adult stem cells and leukemia stem cells (124–126). Studies have confirmed that the occurrence and development of various human tumors, such as leukemia, breast cancer, gastric cancer, lung cancer, bladder cancer, colorectal cancer and esophageal cancer, are related to abnormal expression of the BMI1 gene. BMI1-knockout macrophages exhibit reduced proliferation ability and suppressed expression of angiogenic factors, leading to the loss of their ability to protect MM cells from chemotherapy-induced cell death (123). The Hedgehog-c-Myc axis regulates BMI1 expression, and activation of the hedgehog signaling pathway leads to excessive c-Myc expression in macrophages, thereby promoting BMI1 expression. Upregulation of BMI1 activates survival signals, such as proangiogenic and chemotherapeutic resistance signals, in MM cells (123).

## Macrophages in lymphoma

### Classic Hodgkin lymphoma

Initially, researchers identified macrophage-expressed genes associated with adverse clinical outcomes in patients with advanced classical Hodgkin lymphoma (CHL) through gene expression analysis (127). These genes included ALDH1A1, LYZ, and STAT1. STAT1-positive macrophages were closely related to adverse prognosis, and the expression of macrophage markers, such as LYZ and ALDH1A1, suggested that TAMs play an important role in this process. However, the functional interplay between macrophages and their impact on treatment outcomes remains elusive. Steidl et al. initially demonstrated that an increased abundance of CD68^+^ macrophages was significantly associated with unfavorable outcomes in CHL (128). Furthermore, they found that the clinical relevance of CD68^+^ macrophage content extended to both initial diagnosis and relapse cases, making it a valuable predictor for outcome following primary and secondary interventions.

Subsequently, the role of TAMs in lymphoma has attracted widespread attention. These findings align with previous findings demonstrating that Hodgkin Reed-Sternberg (HRS) cells can stimulate the polarization of macrophages towards the tumor-promoting M2 phenotype through cytokine secretion (129). Conversely, M2-type TAMs support the survival of HRS cells. Currently, the majority of studies have indicated a positive correlation between CD68^+^ or CD163^+^ TAMs and shorter overall survival in patients (130–132), although certain studies have failed to replicate these findings (133, 134). Besides, high levels of CD206 may lead to stromal remodeling and lymphoma dissemination (135).

Interestingly, other studies have demonstrated that a moderate number of macrophages is associated with favorable outcomes in most cases, while patients with either insufficient or excessive macrophages exhibit poorer outcomes (136). Therefore, the authors propose the “excitatory hypothesis” of TAMs, suggesting that a relative deficiency of TAMs may promote HL growth, whereas an increasing number of macrophages demonstrate inhibitory effects. However, beyond a certain threshold, TAMs may once again support tumor growth.

Further studies have shown that TAMs can express programmed death-ligand 1 (PD-L1) (137, 138). These PD-L1 positive macrophages co-localize with HRS cells and closely interact with PD-1 positive T cells, thereby facilitating immune evasion and promoting tumor progression through binding to PD-1 molecules on T cells and NK cells. HL-associated macrophages also express indoleamine 2,3-dioxygenase 1 (IDO-1), which is a tryptophan catabolizing enzyme that inhibits effector T cells and enhances regulatory T cells activity (139). A high proportion of CD68^+^ PD-L1^+^ and CD68^+^ IDO-1^+^ TAMs is associated with a poor prognosis, while the presence of PD-L1^+^ tumor cells, total TAMs, PD-L1^-^ TAMs, or IDO-1^-^ TAMs does not affect the prognosis. These findings further support the notion that in CHL, the adverse prognostic impact of TAMs is linked to immune checkpoints.

### Non-Hodgkin lymphoma

Similar to other hematological malignancies, CD163^+^ TAMs are linked to poor prognosis in diffuse large B-cell lymphoma (DLBCL), follicular lymphoma (FL), cutaneous T-cell lymphoma (CTCL) and splenic marginal zone lymphoma (SMZL) (140–144). There is a positive correlation between CD163^+^ TAMs and the CD163/CD68 ratio in relation to the clinical outcome of DLBCL (145). An increase in the number of CD163^+^ macrophages can promote angiogenesis and is associated with an unfavorable prognosis in FL and DLBCL (146–148). Additionally, the presence of CD204^+^ macrophages in patients with refractory malignant lymphoma undergoing allogeneic hematopoietic cell transplantation was found to be associated with an unfavorable prognosis, which is potentially due to the immunosuppressive properties of these macrophages leading to impaired effector T-cell function and diminished graft-versus-lymphoma effects (149).

In NHL, including FL, MZL, MCL, Burkitt lymphoma and DLBCL, PD-L1 expression is primarily observed in macrophages and rarely on tumor B cells (150–152). This finding is consistent with the observations made in CHL (138, 139). Although the negative impact of PD-L1 on prognosis has been established in solid tumors, McCord et al. suggested that PD-L1 may be associated with a better prognosis in newly diagnosed DLBCL patients receiving chemo-immunotherapy (150). This could be attributed to differences in patient populations or more complex biological processes involving PD-L1 within this heterogeneous disease. High infiltration of PD-1 positive lymphocytes in tumor tissues is associated with a favorable prognosis in patients with FL (152). Patients with lower levels of PD-1positive cells face an increased risk of early transformation into DLBCL, which leads to shortened overall survival.

In conclusion, these reports underscore the intricate interactions between lymphoma cells and TAMs within the immune microenvironment, unveiling the heterogeneity of macrophages and their distinct roles in lymphoma. By comprehending these interactions and underlying mechanisms, novel targeted therapeutic strategies can be developed to specifically target the tumor microenvironment in patients with relapsed or refractory lymphoma.

## MSC-mediated immunoregulation of macrophages

**Figure 2:**
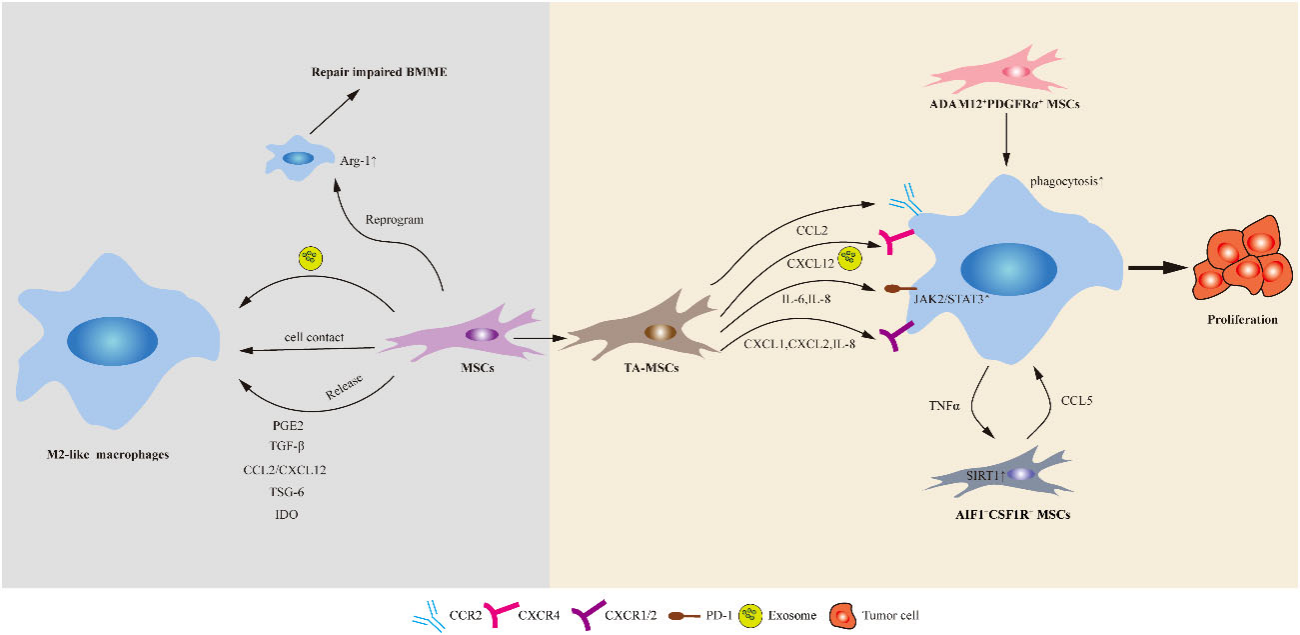
Schematic of the potential mechanisms of action mediated by MSCs on macrophages. MSCs can polarize macrophages via direct cell–cell contact or indirectly, such as through soluble factors and extracellular vesicles (including exosomes). In addition, healthy MSCs implantation could induce the activation of Arg-1^+^ macrophages in the bone marrow of ALL mice and repair damaged BMME. TA-MSCs play a crucial role in promoting tumor growth by recruiting monocytes/macrophages. Two distinct subpopulation of MSCs, namely AIF1^+^CSF1R^+^ MSCs and ADAM12^+^PDGFRα^+^ MSCs, respectively promote macrophage recruitment or enhance macrophage phagocytosis, and induce macrophage polarization towards immunosuppressive phenotypes, thereby driving tumor progression.

The TME plays an important role in the occurrence and development of tumors. Recently, Visser et al. have provided comprehensive insights into the fundamental composition of the tumor microenvironment, its functional regulators, and the dynamic evolution process during cancer development (153). Notably, MSCs and TAMs represent two cell types within the TME that exhibit plasticity and heterogeneity (3, 44). MSCs exert immunomodulatory effects on macrophages through direct cell-cell interactions, paracrine signaling mechanisms, as well as exosome-mediated communication (20, 154). Conversely, macrophages reciprocally regulate the functionality of MSCs (155, 156). The present article provides a comprehensive overview of the role of macrophages in different hematological malignancies and explores the intricate interplay between mesenchymal stem cells and macrophages to elucidate their crucial roles in the occurrence and sustained progression of malignant tumors (**Figure 2**).

Research on the immunomodulatory function of MSCs began in 2002 (157, 158). In that year, two teams independently discovered the powerful immunosuppressive ability of MSCs. This discovery has since led to progress in MSCs therapy in immune diseases, thus fueling researchers’ efforts to delve more deeply into the immunomodulatory function of MSCs. Under the stimulation of inflammatory cytokines, MSCs are capable of expressing various immunosuppressive factors, including prostaglandin E2 (PGE2), TGF-β, CCL2/CXCL12, TNF-stimulated gene 6 (TSG-6) and IDO, as well as induce macrophage polarization towards the anti-inflammatory M2 phenotype (159, 160).

In 2009, it was discovered that MSCs induce macrophage polarization through the secretion of PGE2 (161). This polarization is characterized by a significant increase in IL-10 secretion, while the circulating levels of TNF-α and IL-6 are markedly decreased. Subsequently, studies have revealed that MSCs rely on the COX2-PGE2 pathway to regulate monocyte differentiation into macrophages and polarize them towards an M2 phenotype (with increased expression of CD163 and CD206) (162, 163). This process inhibits the production of pro-inflammatory factors such as TNF-α and IL-17 while promoting the release of anti-inflammatory factors such as IL-10 and TGF-β (163, 164). Ultimately, this effect is achieved by suppressing effector immune cells and inducing regulatory T cells (Tregs) to suppress immune responses (165).

TGF-β is another important immunosuppressive factor secreted by MSCs that can induce macrophage M2 polarization and inhibit immune responses (166). MSCs-derived TGF-β downregulated the expression of M1 markers, upregulated the expression of M2 markers, and attenuated excessive activation of the inflammatory response. A recent study revealed the presence of a unique type of MSCs (known as slow-cycling ADAM12^+^PDGFRα^+^ MSCs) at tumor edges in melanoma, pancreatic cancer, and prostate cancer mouse models (167). These MSCs promote macrophage efferocytosis and polarization through TGF-β signaling, thereby inducing pathological angiogenesis and immune suppression.

BM-MSCs secrete a large number of chemokines, including CCL2 and CXCL12, which are the main chemotactic factors for monocytes and macrophages (168–170). CCL2 plays a crucial role in regulating the migration and invasion of monocytes/macrophages (168). Whelan et al. discovered that wild-type MSCs can induce polarization of M2 macrophages in mouse wounds through CCL2, resulting in reduced inflammation, enhanced epithelial regeneration, and accelerated wound healing (171). Besides, BM-MSCs can secrete CCL2 and CXCL12, which form heterodimers that promote macrophage polarization towards the M2 phenotype. This process also mobilizes IL-10^+^ T cells and B cells in the intestine, resulting in a suppressive effect on colitis (172).

MSCs exposed to inflammation are also capable of secreting TSG-6, which is a multifunctional protein with anti-TNFα release, anti-inflammatory, and tissue protective properties (173, 174). Choi H demonstrated for the first time that TSG-6 inhibits the activation of the NF-κB signaling pathway within macrophages by binding to the CD44 receptor on their surface, thereby weakening the pro-inflammatory signals initiated by macrophages (175). TSG-6 derived from MSCs induces macrophage polarization in inflammatory bowel disease (IBD) mice, which promotes the release of more M2 macrophages into the colon and suppresses inflammatory responses (176). In general, TSG-6 is capable of reducing the number of M1 macrophages and promoting their transformation to the M2 phenotype (177).

Similarly, IL-6 also has similar regulatory functions on macrophages (178). Specifically, AML-MSCs secrete a substantial quantity of IL-6 and induce epithelial-mesenchymal transition (EMT)-like transformation in AML cells through the Jak2/Stat3 signaling pathway, potentially contributing to chemotherapy resistance (179). Additionally, IL-6/Jak2/Stat3 signaling is involved in the M2 polarization of macrophages, thereby promoting brain metastasis in non-small cell lung cancer (180). Furthermore, IL-6 can stimulate TAMs to produce CCL-20 and attract CCR-6-expressing B cells and γδ T cells for aggregation, ultimately driving colorectal cancer progression (181).

In addition to the traditional paracrine mode, cell-cell contact and vesicle transfer are also pathways through which MSCs trigger M2 anti-inflammatory phenotypes and exert immune regulatory functions (182–185). Extracellular vesicles (EVs) have been shown to play an important role in intercellular communication. EVs (including exosomes) can transfer many soluble cytokines and molecules, (including proteins, nucleic acids and organelles), from donor cells to recipient cells and can change the protein expression of target cells (186, 187). Accumulating evidence suggests that MSCs-derived exosomes play an important role in immune regulation. Breast cancer-derived MSCs can induce monocyte myeloid-derived suppressor cells to differentiate into M2-polarized macrophages through exosomes and increase their immunosuppressive activity, thereby accelerating malignant progression (188). Furthermore, MSCs-derived exosomes have been extensively investigated in many preclinical studies of inflammatory diseases. In a study of inflammatory bowel disease (IBD), exosomes derived from MSCs attenuated colonic mucosal inflammation by polarizing macrophages to an M2b-like phenotype with increased IL-10 production and secretion (189). Similarly, MSC-derived exosomes have the ability to induce a phenotypic shift in macrophages from pro-inflammatory to anti-inflammatory, thereby attenuating inflammation in conditions such as myocardial injury (190), retinal injury (191), spinal cord injury (192), and lung injury (193).

## Therapeutic potential of targeting MSCs or macrophages

Numerous research findings have consistently demonstrated the safe and efficacious role of MSCs in the treatment of various diseases, including graft-versus-host disease, autoimmune disorders, cardiovascular diseases, as well as bone and cartilage injuries (4, 194). The therapeutic principles underlying these stem cells primarily involve their potential for multidirectional differentiation, paracrine effects, immune modulation, and intercellular substance transfer. Currently, the treatment strategies for MSCs primarily include the following (4): 1) utilizing MSCs as carriers for anticancer drugs and genetically modifying them to express or secrete various tumor-inhibiting agents; 2) disrupting paracrine signaling pathways to impede the interaction between MSCs and tumor cells or immune cells; 3) reversing the pro-tumor characteristics of TA-MSCs while synergistically enhancing therapeutic effects with chemotherapy drugs; and 4) modifying exosomes derived from MSCs to accomplish cell-free tumor therapy.

Macrophages also play dual roles in the TME and represent complex and pivotal targets for immunotherapy. Currently, extensive research is being conducted on macrophages, with a focus on four primary strategies: eliminating TAMs, inhibiting TAMs recruitment processes, repolarizing TAMs to the M1 phenotype to augment their anti-tumor functions, and targeting TAMs regulation. The role of macrophages in tumors and their therapeutic potential were reviewed in more detail by Li et al. (44) and Kloosterman et al. (40).

As mentioned above, CCL2 is an important regulatory factor in the recruitment of macrophages and is involved in several key steps of tumor formation and metastasis, including promoting angiogenesis, recruiting myeloid-derived suppressor cells, regulating cancer cell invasiveness, and inducing pro-survival signals in different cancer cells (168, 172, 195). MSCs-derived CCL2 plays a crucial role in the M1-M2 polarization of macrophages, which is also a key feature of the therapeutic response of MSCs. Compared to BM-MSCs, TA-MSCs exhibit significantly elevated levels of CCL2 and can recruit TAMs to enhance primary tumor growth, invasiveness, and metastatic speed. Therefore, the MSCs-CCL2-monocyte axis has significant physiological significance in tumor progression, and the blockage of this interaction may be an effective strategy for targeted cancer treatment based on MSCs.

The CXCL12/CXCR4 signaling pathway plays an important role in maintaining normal hematopoiesis, and is essential for the homing and bone marrow residence of AML and ALL cells (196, 197). TA-MSCs can secrete a large amount of CXCL12 to activate the overexpressed specific receptor CXCR4 on AML cells, thereby guiding AML cells to migrate to the BMME and acquire drug resistance signals (196). The CXCR4-CXCL12 axis also promotes TAMs differentiation into perivascular TAMs, increases vascular permeability, and allows tumor cells to infiltrate blood vessels (198). In addition, MSCs-derived CXCL12 can induce BMDMs to transform into M2 phenotype, thus forming an immunosuppressive microenvironment that enables malignant cells to evade immune surveillance and promoting tumor growth and metastasis (198). It has been proven that using CXCR4 antagonists can alter the interaction between tumors and stroma, increase cancer cell sensitivity to cytotoxic drugs, and alleviate the burden of tumor growth and metastasis.Recently, researchers have identified a pro-inflammatory subset of MSCs (referred to as AIF1^+^CSF1R^+^MSCs) in rats with primary liver cancer (199). This particular subset is present throughout the entire process of liver cancer development. The findings from this study demonstrate that TNF-α derived from macrophages upregulates sirtuin 1 expression in MSCs. Conversely, educated MSCs secrete CCL5 to facilitate macrophage recruitment and establish a chronic inflammatory microenvironment that drives the occurrence of hepatocellular carcinoma (HCC). Depletion of macrophages or knockdown of CCL5 weakens the promoting effect of MSCs on hepatic inflammation and HCC occurrence, indicating that the role of MSCs in HCC relies on infiltration by macrophages. These discoveries enhance our understanding of the immunomodulatory effects exerted by MSCs and shed light on the mechanisms underlying liver cancer development. Furthermore, these findings enrich our knowledge regarding the interaction between MSCs and macrophages, providing valuable insights for targeted therapy strategies involving stromal cells.

In addition, in various solid tumors, such as melanoma, a population of slow-cycling ADAM12^+^PDGFRα^+^ MSCs exists (167). These cells facilitate macrophage phagocytosis by upregulating the gene expression of Gas6, Lgals and Csf1 and induce macrophage polarization towards an immunosuppressive and pro-angiogenic phenotype, thereby driving tumor progression. TGF-β plays a pivotal role in this process by inducing the expression of ADAM12. The specific knockout of TGF-βR2 in ADAM12^+^ cells significantly impeded tumor growth while enhancing T-cell infiltration and promoting vascular normalization. This further underscores the regulatory influence of slow-cycling ADAM12^+^MSCs on the tumor microenvironment through the TGF-β signaling pathway.

In addition to modifying TA-MSCs for therapy, non-gene-edited healthy MSCs can also be utilized in the treatment of leukemia. Recent research suggests that umbilical cord MSCs promote AML cell differentiation and apoptosis through the transfer of vesicles rich in neutrophil elastase (NE), thereby exerting an anti-AML effect (200). Activation of the vitamin D receptor (VDR) plays a crucial role in NE release within vesicles. The combined administration of MSCs and 1,25D3 (a VDR activator) synergistically enhances AML cell differentiation, reduces leukemia burden, and significantly improves survival rates in AML mice. Another study showed that healthy MSCs implantation could induce the activation of Arg-1^+^ macrophages in the bone marrow of ALL mice, repair damaged BMME, and delay the progression of leukemia (201). This effect seems to be inconsistent with the immunosuppressive function of MSCs in promoting macrophage-mediated tumor progression, which may be attributed to differences in disease states, sources and injection quantities of MSCs. This study also highlights the complexity of the interplay between MSCs and macrophages in the TME. The abovementioned results provide a new perspective on the use of healthy MSCs for the treatment of leukemia. However, there is currently relatively limited research on the regulatory effects of implanted healthy MSCs on immune cells such as macrophages.

In summary, the primary interaction between MSCs and macrophages is predominantly mediated by secretory factors. Abundant cell-derived factors originating from MSCs possess the ability to modulate the immune function of macrophages and actively participate in disease progression, and vice versa. Due to the complexity and diversity of these processes, further research is imperative to identify novel strategies for stromal cell-based immunotherapy.

## Interaction between MSCs and macrophages in malignancies

In addition to macrophages being reshaped by tumor cells, MSCs can also be transformed into TA-MSCs. These TA-MSCs not only directly interact with tumor cells but also regulate the TME indirectly by interacting with surrounding immune cells, thereby promoting tumor development (202, 203). Currently, there is limited research on the interaction between MSCs and macrophages in hematological tumors. We summarize their interactions in hematological malignancies and discuss their relevant studies in solid tumors (**Figure 2**).

To the best of our knowledge, Ren et al. first identified the interaction between MSCs and immune cells within tumors in 2012, providing evidence that TA-MSCs play a crucial role in promoting tumor growth by recruiting monocytes/macrophages (168). Research has found that lymphoma-derived MSCs (L-MSCs) exhibit a more prominent role in promoting tumor growth compared to BM-MSCs or skin MSCs. In comparison to BM-MSCs, L-MSCs demonstrate significant advantages in recruiting immune cells, specifically increasing the number of CD11b^+^Ly6C^+^ monocytes and F4/80^+^ macrophages in tumors and peripheral blood. Additionally, L-MSCs can induce the polarization of macrophages towards the M2 phenotype, characterized by high levels of IL-10 expression and low levels of IL-2, TNFα, and MHC-II expression. Ultimately, the results revealed that the elimination of monocytes/macrophages could abolish the tumor-promoting effect of L-MSCs. Furthermore, the pro-tumor effect relies on macrophage recruitment and phenotypic switching through the CCL2-CCR2 pathway.

CCL2 is the most significantly differentially expressed gene between FL-MSCs and HD-MSCs and promotes the migration of monocytes to tumor sites (147). FL-MSCs drive monocytes towards TAMs-like differentiation, which promotes angiogenesis and tumorigenic effects, while also synergistically supporting the growth of FL B cells *in vitro* with TAMs. These results demonstrate that CCL2 produced by FL-MSCs facilitates tumor cell growth by attracting monocytes rather than by directly acting on B cells.

In addition, CXCL12 derived from BM-MSCs can induce BMDMs to exhibit M2-like characteristics and inhibit their phagocytic function, thereby promoting the growth of mouse mammary tumors (204). Biswas et al. further confirmed that TA-MSCs from breast cancer release exosomes that are rich in TGF-β, C1q, and semaphorins (188). These exosomes promote the overexpression of PD-L1 in immature myelo-monocytic precursors and CD206^+^ macrophages, stimulating macrophages to produce arginase-1 and IL-10 to suppress anti-tumor immune responses and thereby accelerate tumor growth. Similarly, gastric cancer-derived MSCs (GC-MSCs) activated the M2 polarization of macrophages (increased CD204^+^/CD163^+^ cells) by producing IL-6 and IL-8 to activate the Jak2/Stat3 signaling pathway (205). Consequently, pretreated macrophages enhance the migratory and invasive capabilities of gastric cancer cells while significantly promoting the EMT process.

In the microenvironment of ovarian tumors, BM-MSCs can differentiate into cancer-associated-MSCs (CA-MSCs), leading to carboplatin resistance in ovarian tumor cells (206). However, injecting BM-MSCs into the peritoneal cavity did not inhibit tumor progression in mice but rather suppressed the efficacy of chemotherapy drugs. This observation also suggested that BM-MSCs may be re-educated into CA-MSCs and exert an anti-chemotherapy effect. The study revealed that CA-MSCs highly express CXCR1/2 ligands such as CXCL1, CXCL2, and IL-8, which promote monocyte differentiation towards a pro-tumor M2 phenotype, facilitating tumor progression and the acquisition of chemotherapy resistance. IL-8-CXCR1/2 plays a crucial role in initiating and promoting inflammation-mediated metastasis, as well as tumor growth and dissemination. The inhibition of CXCR1/2 leads to CA-MSCs-mediated loss of the M2 phenotype in these activated macrophages, and these cells exhibit antitumor effects. By utilizing CXCR1/2 inhibitors, the sensitivity of ovarian tumor cells to carboplatin can be restored, thereby counteracting the promoting effect of CA-MSCs on tumors.

Another study confirmed that healthy MSCs are able to reprogram leukemic macrophages (L-Macs) to repair the impaired BMME and inhibit leukemia progression (201). The administration of healthy MSCs to leukemic mice significantly reduced the tumor burden, stimulated megakaryocyte activation, and improved the overall survival rate. *In vitro* co-culture experiments showed that MSCs could significantly up-regulate the expression of Arg1, a marker of tissue repair, in L-Macs, and the expression of Arg1 in macrophages isolated from the bone marrow of MSC-treated leukemia mice was also significantly increased. Ultimately, when co-cultivated macrophages were re-infused into leukemia mice, the quantity of bone marrow MSCs in the mice significantly increased, and the co-cultivated macrophages exhibited similar anti-leukemia effects.

However, the regulatory mechanisms by which TAMs modulate MSC function remain unexplored. After treatment with conditioned medium derived from M1 macrophages, MSCs (cMSCs) exhibited significantly enhanced transcription levels of iNOS, monocyte chemoattractant protein 1 (MCP-1), IL-6, and Cox-2 compared to those in untreated MSCs (156). Conversely, cMSCs promoted tumor cell growth (including breast cancer, hepatocellular carcinoma and glioblastoma) through NO-mediated immunosuppression and MCP-1- and IL-6 -mediated macrophage trafficking and polarization towards an M2-like phenotype. In summary, during the initiation stage of tumors, polarization of M1 macrophages and release of inflammatory factors occur, leading to functional remodeling of surrounding MSCs. Subsequently, these remodeled MSCs tend to transform macrophages into M2-like state and exert immunosuppressive effects that promote tumor growth.

Taken together, these findings highlight the complexity of the interaction between MSCs and macrophages in the TME. Currently, there is a dearth of research on such interactions in hematologic tumors, necessitating further investigation to identify potential therapeutic targets.

## Conclusion

In the past few decades, a large number of therapies based on MSCs have emerged for preclinical research to treat various pathological conditions, including neurological disorders, myocardial ischemia, diabetes, bone and cartilage diseases, as well as tumors. Furthermore, by reprogramming TA-MSCs, their pro-tumorigenic attributes can be effectively reversed.

However, increasing evidence suggests that there are interactions between MSCs and tumor cells that can result in both the promotion and inhibition of tumor development. These discrepancies may arise from differences in experimental tumor models, sources of MSCs, dosage or timing of MSCs treatments, methods of cell delivery, choice of control groups, and other experimental conditions. In addition to directly affecting tumor cells, MSCs also play an important immunomodulatory role and indirectly participate in the pathological processes of tumors.

In summary, as major components of the tumor microenvironment, both MSCs and macrophages can be remodeled and exhibit different phenotypes and functions during tumor initiation and progression. However, in hematological malignancies, the cellular and molecular mechanisms underlying the interaction between MSCs and macrophages have not been clearly elucidated. The immunosuppressive effects of MSCs are still under investigation, thus necessitating further exploration of the reciprocal interactions between MSCs and macrophages to provide better therapeutic strategies.

## Author contributions

KL: Funding acquisition, Writing – original draft. HN: Supervision, Writing – original draft. RJ: Writing – review & editing. XW: Funding acquisition, Writing – review & editing.
